# Editorial: B-cell engineering in malignant and non-malignant disease

**DOI:** 10.3389/fimmu.2026.2014160

**Published:** 2026-10-09

**Authors:** Lucia Sereni, Valentina Capo, Francesca Pala

**Affiliations:** 1 Dana-Farber Cancer Institute, Pediatric Oncology, Boston, MA, United States; 2 Boston Children’s Hospital, Hematology/Oncology Division, Boston, MA, United States; 3 Harvard Medical School, Boston, MA, United States; 4 San Raffaele Telethon Institute for Gene Therapy (SR-Tiget), IRCCS San Raffaele Scientific Institute, Milan, Italy; 5 Milan Unit, Institute of Genetic and Biomedical Research, Consiglio Nazionale delle Ricerche, Milan, Italy; 6 National Institute of Allergy and Infectious Diseases (NIH), Bethesda, MD, United States

**Keywords:** antibodies, B cell lymphoma, B cells, genome engineering, pre-eclampsia, vasculitis

Advances in genome engineering and gene delivery have substantially expanded the use of B cells (1, 2). Early work introduced predefined antibody specificities or used B cells and plasma cells as vehicles for sustained protein secretion. Precise editing of the endogenous immunoglobulin loci now allows engineered B cells to acquire a chosen specificity while retaining regulated BCR expression and antibody secretion — and, critically, the cell’s own biology: antigen responsiveness, germinal-center participation, somatic hypermutation and affinity maturation. The introduced therapeutic function can therefore evolve after the cells are administered.

This shifts B-cell engineering from delivering a fixed product toward programmable, antigen-responsive and potentially self-amplifying therapeutics. The same principle has been extended to hematopoietic stem and progenitor cells, whose edited B-cell progeny produce therapeutic antibodies durably and can be boosted by antigen exposure (3). B-cell engineering may thus combine the durability of stem-cell therapy, the specificity of antibody therapeutics, and the amplification and adaptive properties of vaccination.

This versatility creates opportunities across a remarkably broad spectrum of diseases. In infectious diseases, engineered B cells could provide sustained production of protective antibodies while vaccination can drive their expansion and antibody affinity. In cancer, B cells may be programmed to produce tumor-targeting or immunomodulatory molecules, present tumor antigens, or acquire chimeric antigen receptors. For the treatment of immune-mediated disorders, B cell capacity for long-term protein secretion and immune regulation can be exploited.

However, efficient and selective gene delivery, preservation of B-cell fitness and differentiation, genomic integrity, control of therapeutic protein expression, persistence and trafficking, and scalable manufacturing remain important challenges in B-cell engineering. The development of increasingly precise genome-editing technologies together with improved viral and non-viral delivery platforms, including strategies for direct *in vivo* engineering, is expected to further broaden the therapeutic potential of this approach.

Against this rapidly evolving background, the Research Topic “B-cell Engineering in Malignant and Non-Malignant Disease” brings together five contributions that illustrate how advances in genome engineering, antibody design and cellular immunotherapy are transforming B cells from targets of therapeutic intervention into versatile cellular platforms that can be programmed to recognize, respond, adapt and deliver therapeutic functions.

Two original research contributions apply this concept to solid tumors, and together they argue that an engineered B cell is best understood as a multi-modal effector rather than an antibody-delivery vehicle. Guberman Bracha et al. edited the IgH locus of primary mouse B cells with CRISPR-Cas9 ribonucleoproteins and rAAV-delivered donor cassettes, so that an anti-HPV-E6 specificity is displayed as a membrane BCR, secreted upon differentiation into antibody-secreting cells, and remains a substrate for somatic hypermutation. Boucher et al. extended the platform to primary human B cells and to two classes of target — the intracellular oncoprotein E6 and the membrane oncofetal antigen claudin-6 (CLDN6). Engineered human B cells expressed signaling-competent BCRs, differentiated to switched-memory and plasmablast phenotypes, and secreted antigen-specific IgG that mediated ADCP, CDC and ADCC against CLDN6-positive but not CLDN6-negative tumor lines, while also acquiring tumor-derived antigen and presenting it to E6-specific T cells. Both studies are candid about the obstacles ahead: low or heterogeneous antigen expression, antigen shedding, transgenic–endogenous chain mispairing, the genotoxic and translocation risks of nuclease-mediated IgH editing, and the need for larger-animal models in which these cooperative mechanisms can be properly evaluated.

The third contribution turns from engineering B cells to modeling what happens when B cells become malignant. Yuan and Wang review genetically engineered mouse models (GEMMs) of diffuse large B-cell lymphoma,. Their central message is that a single mutation is rarely enough to transform a B cell: faithful models need several cooperating lesions, which is why the field increasingly combines germline alleles with CRISPR-Cas9 editing or retroviral oncogene delivery in bone-marrow chimeras. They argue, equally, that such models must be immunocompetent, because central features of lymphoma biology are invisible in immunodeficient xenograft hosts.

Two contributions in this Topic examine settings in which B-cell biology, left unchecked, becomes a driver of non-malignant disease rather than a tool against it. Shimojima et al. review the role of BAFF and APRIL signaling in ANCA-associated vasculitis, showing that persistently elevated BAFF/APRIL levels — and altered expression of their receptors across B-cell subsets — help explain why autoreactive B cells and ANCA production so often re-emerge after rituximab-induced remission, and why combination strategies targeting BAFF/APRIL alongside B-cell depletion are now under active clinical investigation. Nair et al. take a parallel, integrative approach to pre-eclampsia, proposing that toll-like receptor activation, pathogenic B1a cells producing agonistic autoantibodies against the angiotensin II type 1 receptor, and feto-maternal microchimerism do not act independently but form a self-reinforcing immunological circuit that sustains inflammation and impairs placental angiogenesis. Together, these two reviews illustrate a shared lesson: in non-malignant disease, pathology often arises from B-cell abnormal persistence, phenotypic switching, or misdirected specificity — the same molecular handles that engineered B-cell therapies elsewhere in this Topic aim to control deliberately.

What unites these five contributions is the B-cell receptor and the antibody it encodes. In the two oncology studies, it is rewritten deliberately to give a B cell a new target. In the lymphoma models, it is a dependency the tumor cannot survive without. In vasculitis and pre-eclampsia, it goes wrong on its own, generating antibodies directed at self. The therapeutic question is therefore the same: which antibody specificities persist, how strongly they signal, and what the cell does in response. This is where the two fields could borrow from each other. The methods used to install a chosen specificity at the IgH locus might equally be used to replace an autoreactive one, rather than deleting the cell that carries it; conversely, what autoimmunity has learned about BAFF and APRIL bears on whether engineered B cells will persist after transfer. The problem is a single one approached from opposite ends: how to control which antibody specificities a B cell carries, and how long they last. Progress will come faster if the fields that engineer B cells and those that study their dysregulation read each other more closely.
